# Transcriptomic Profiling of Mouse Mast Cells upon Pathogenic Avian H5N1 and Pandemic H1N1 Influenza a Virus Infection

**DOI:** 10.3390/v14020292

**Published:** 2022-01-29

**Authors:** Yuling Tang, Hongping Wu, Caiyun Huo, Shumei Zou, Yanxin Hu, Hanchun Yang

**Affiliations:** 1 Key Laboratory of Animal Epidemiology of Ministry of Agriculture, College of Veterinary Medicine, China Agricultural University, Beijing 100193, China; YulingTang1868@163.com (Y.T.); hongpingwu@mail.tsinghua.edu.cn (H.W.); yanghanchun1@cau.edu.cn (H.Y.); 2 Beijing Key Laboratory for Prevention and Control of Infectious Diseases in Livestock and Poultry, Institute of Animal Husbandry and Veterinary Medicine, Beijing Academy of Agriculture and Forestry Sciences, Beijing 100097, China; zhongquanbagong01@163.com; 3 Collaboration Innovation Center for Diagnosis and Treatment of Infectious Diseases, Chinese Center for Disease Control and Prevention, Key Laboratory for Medical Virology, National Health and Family Planning Commission, National Institute for Viral Disease Control and Prevention, Beijing 100013, China; zoushumei@163.com

**Keywords:** influenza A virus, Nbeal2, mast cell, transcriptome

## Abstract

Mast cells, widely residing in connective tissues and on mucosal surfaces, play significant roles in battling against influenza A viruses. To gain further insights into the host cellular responses of mouse mast cells with influenza A virus infection, such as the highly pathogenic avian influenza A virus H5N1 and the human pandemic influenza A H1N1, we employed high-throughput RNA sequencing to identify differentially expressed genes (DEGs) and related signaling pathways. Our data revealed that H1N1-infected mouse mast P815 cells presented more up- and down-regulated genes compared with H5N1-infected cells. Gene ontology analysis showed that the up-regulated genes in H1N1 infection were enriched for more degranulation-related cellular component terms and immune recognition-related molecular functions terms, while the up-regulated genes in H5N1 infection were enriched for more immune-response-related biological processes. Network enrichment of the KEGG pathway analysis showed that DEGs in H1N1 infection were specifically enriched for the FoxO and autophagy pathways. In contrast, DEGs in H5N1 infection were specifically enriched for the NF-κB and necroptosis pathways. Interestingly, we found that Nbeal2 could be preferentially activated in H5N1-infected P815 cells, where the level of Nbeal2 increased dramatically but decreased in HIN1-infected P815 cells. Nbeal2 knockdown facilitated inflammatory cytokine release in both H1N1- and H5N1-infected P815 cells and aggravated the apoptosis of pulmonary epithelial cells. In summary, our data described a transcriptomic profile and bioinformatic characterization of H1N-1 or H5N1-infected mast cells and, for the first time, established the crucial role of Nbeal2 during influenza A virus infection.

## 1. Introduction

Influenza A viruses (IAVs) are a class of aggressive pathogens that lead to acute respiratory infections in humans and animals and result in periodic pandemics around the world, thus, posing a great threat to human and animal health due to their potential rapid spread and high morbidity and mortality [1,2]. IAVs are divided into different subtypes on the basis of the surface viral glycoproteins hemagglutinin (HA) and neuraminidase (NA). H5N1 is considered a highly pathogenic avian influenza virus, characterized by the robust immune response known as a “cytokine storm” during infection [3,4], while the 2009 pandemic IAV H1N1 is a new strain of influenza virus that causes common flu symptoms.

Mast cells (MCs) are an important effector and regulatory immune cells in the body, widely distributed in the mucosa and submucosal connective tissues of the skin, uterus, bladder, respiratory tract and gastrointestinal tract [5]. MCs are located around blood vessels and nerves forming the body’s first line of defense against infection. They are very sensitized to immune and non-immune stimuli that widely exist in the external environment to make rapid responses [6].

Previous studies on MCs mainly focused on allergic diseases. In recent years, immune regulation of MCs in the process of neoplastic disease, parasite, bacterial and virus infection has attracted much attention [7,8,9]. Our previous studies indicated that H1N1 and H5N1 viruses replicate effectively in P815 cells and that H1N1 virus shows more efficient replication [10]. However, the detailed molecular mechanisms by which MCs respond to H1N1 and H5N1 infection remain unclear.

High-throughput RNA sequencing (RNA-seq) technology coupled with bioinformatic analysis has revolutionized the study of biomedical research in recent years. Cellular signaling mechanisms born out of transcriptomic analysis can uncover cellular and metabolic alterations and reveal the pathogenesis of diseases. For instance, the PI3K–AKT /mTOR pathway was reported to regulate adipocyte differentiation as determined by KEGG enrichment analysis [11]. The correlation between cellular signaling, such as TLR3 and interferon signaling pathways, and the expression of mediators of inflammation was also reported [12].

Not surprisingly, transcriptomic analysis has been widely adopted to study defense mechanisms against virus infection with the hope to identify interactions between the host and influenza viruses [13]. Over the past two decades, a number of transcriptomic studies on IAVs and host responses have been published [14,15,16,17,18]. As viral pathogenesis pertains the interactions between host responses and viruses, thoroughly understanding the key host molecular driver(s) and viral gene effector(s) is expected to lay the foundation for curing these diseases [15].

The differences in the pathogenesis observed between different IAV strains are complicated with characteristics specific to virus strains and the host immune responses [16]. In order to identify the differences in cellular responses against two IAV strains, we studied the host immune responses of mouse MCs and P815 cells infected with H1N1 virus and H5N1 virus using RNA-seq. The identification, quantification and functional annotation of differentially expressed genes (DEGs) were carried out by gene ontology (GO) enrichment analysis and KEGG pathway analysis.

GO annotation revealed specific GO terms of molecular function (MF), biological process (BP) and cellular component (CC). KEGG pathway analysis searched for related molecular interactions and relationship networks among metabolism, biochemical signaling, regulation and diseases. In particular, we focused on how the Neurobeachin-like 2 Protein (Nbeal2) was induced by the two subtypes of IAV infection in order to provide novel insights into its potential mechanism mediating pathogenesis.

To the best of our knowledge, our research established, for the first time, that Nbeal2 of mouse MCs may play a significant role in the inflammatory response upon influenza A virus infection.

## 2. Materials and Methods

### 2.1. Viruses and Cell Culture

The H1N1 (A/WSN/33) and H5N1 (A/Chicken/Henan/1/04) viruses used were described in our previous publication, and virus titers were determined as described previously [19,20]. The mouse mastocytoma cell line P815 was provided by the Cell Resource Center of Peking Union Medical College (Beijing, China) and cultured in Dulbecco’s minimal essential medium (DMEM; HyClone Laboratories, Logan, UT, USA, SH30022.01) containing 10% fetal bovine serum (FBS; HyClone), 100 U/mL penicillin and 100 μg /mL streptomycin. All experiments concerned with IAVs were carried out in a biosafety level 3 containment laboratory approved by the Ministry of Agriculture of China.

### 2.2. In Vitro Viral Infection

P815 cells were grown in 6-well (1 × 10^6^ cells/well) plates and incubated at a multiplicity of infection (MOI) of 1 for 1 h at 37 °C. Cell monolayers were washed with phosphate buffer saline (PBS) and then incubated with DMEM supplemented with 1% FBS for 12 h.

### 2.3. RNA Extraction

The total RNA of P815 cells was extracted at 12 h post-infection using Trizol reagents (Invitrogen, Life Technologies, Grand Island, NY, USA) according to the manufacturer’s instructions.

### 2.4. cDNA Preparation and Next Generation Sequencing

The RNA concentration was determined using a NanoDrop 2000 (Thermo-Fisher Scientific, Waltham, MA, USA, ND2000), and RNA quality was assessed with an Agilent 2100 Bioanalyzer (Agilent, Santa Clara, CA, USA, Agilent 2100). Viral RNA treated with DNase was reversely transcribed into cDNA. RNA-seq was performed by the Illumina Novaseq6000 to generate 150-bp paired-end reads. Biological triplicates of each group were used and analyzed in this study. The raw reads were cleaned by removing the adaptor sequence and low-quality reads. 

The cDNA libraries were prepared according to the standard Illumina protocol. To annotate and evaluate the transcript abundances from the sequenced reads, the reference genome file: GCF_000001635.26_GRCm38.p6_genomic.fna.gz (download link: https://ftp.ncbi.nlm.nih.gov/genomes/all/GCF/000/001/635/GCF_000001635.26_GRCm38.p6/GCF_000001635.26_GRCm38.p6_genomic.fna.gz, accessed on 20 December 2021) and reference genome annotation file: GCF_000001635.26_GRCm38.p6_genomic.gff.gz (download link: https://ftp.ncbi.nlm.nih.gov/gnomes/all/GCF/000/001/635/GCF_000001635.26_GRCm38.p6/GCF_000001635.26_GRCm38.p6_genomic.gff.gz, accessed on 20 December 2021) were used as references. 

After the mouse genome index file was established with clean reads using HISAT2 software, mapping the sequencing reads to the reference genome efficiently according to the gene location information specified in the genome annotation file *. gtf was carried out. DESeq2 software was used for screening DEGs, and the screening criteria were set as |log2FoldChange| > 1 and FDR-adjusted *p*-value (adjp) < 0.05. 

The online website g: Profiler, which is an open-access resource available at https://biit.cs.ut.ee/gprofiler/gost, accessed on 20 December 2021, was used for the functional enrichment analysis of DEGs. The 30 subsets of enriched KEGG pathway terms with the significant p-values were selected and rendered as a network plot. The network was visualized using Cytoscape, where each node represents an enriched term and is labeled by its cluster ID and p-value with color mark.

### 2.5. Confirmation of DEGs by Quantitative Real-Time Polymerase Chain Reaction (qRT-PCR) Analysis

To validate the differential expression of selected DEGs obtained by RNA-seq analysis and their significant roles in inflammatory responses, qRT-PCR was performed. The procedures were as previous described [21].

### 2.6. RNA Interference (RNAi)

Three independent siRNAs for the mouse *Nbeal2* gene were designed and provided by GenePharma (Suzhou, China). We seeded 1 × 10^6^ P815 cells in 6-well plates, and transfection was carried out when cultures were 70% confluent. We diluted 5 µL Lipofectamine 2000 reagent (Invitrogen, Carlsbad, CA, USA, 11668019) in 245 µL DMEM and incubated at room temperature for 5 min. 

Additionally, 100 pmol siRNA duplex or negative control siRNA was diluted in 250 µL DMEM, mixed with pre-diluted Lipofectamine 2000 for 20 min at room temperature and then was added into the matching well. The transfection efficiency was measured by qRT-PCR. The sequences were listed in Table 1.

### 2.7. Western Blotting

Cells were harvested at the 24 h after infection and lysed with RIPA lysis buffer containing protease inhibitor cocktail (Beyotime Institute of Biotechnology, Beijing, China). Protein concentrations were determined using a BCA protein assay kit (Beyotime Institute of Biotechnology). Equal amounts of protein were separated on 10% SDS-PAGE gel and transferred to a polyvinylidene difluoride (PVDF) membrane (Millipore, Beijing, China). 

The membranes were blocked using 5% non-fat dry milk (BD Biosciences, Franklin Lakes, NJ, USA) at room temperature for 2 h, washed and probed using the specified antibodies. Nbeal2 rabbit mAb (bs-1903R, Bioss, Beijing, China) and β-actin mouse mAb (K200058M, Solarbio, Beijing) were used, and corresponding horseradish-peroxide-conjugated secondary antibodies were obtained from Beyotime (Beijing, China). Protein bands were visualized using Western Lightning Plus-ECL (Perkin Elmer, Waltham, MA, USA). β-actin served as a loading control.

### 2.8. Analysis of A549 Cell Apoptosis by Flow Cytometry

P815 cells were infected with H1N1 and H5N1 viruses (1 pfu/cell). The supernatants were collected at 24 h post-infection and prepared by ultracentrifugation for 3 h at 30,000 rpm to remove viral particles. There was no viral RNA or infectious viral particle detected in the supernatants by qRT-PCR or haemagglutination assays. A549 cells (a human lung cell line) were incubated with the supernatant, and apoptosis was measured 24 h later by flow cytometry using ANNEXIN V-FITC/PI (Solarbio, Beijing China) as previously described [22].

### 2.9. Data Statistics and Analysis

GraphPad Prism V8.0 software was used to analyze the statistical graphs. Data are shown as the mean ± SD. Statistical analysis was performed by two-way analysis of variance (ANOVA) contained in the prism software suite. The results are expressed as the means and standard deviations.

## 3. Results

### 3.1. Gene Transcription Profile of Mast Cells upon IAV Infection

**Figure 1:**
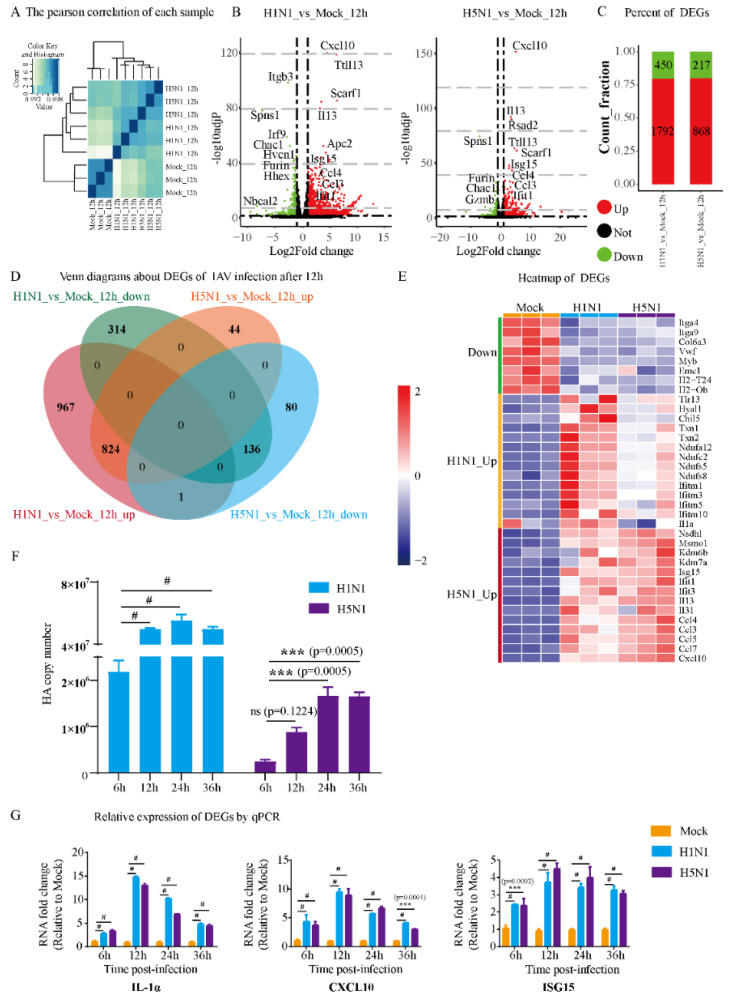
Analysis of DEGs and verification of dynamic patterns of gene expression in H1N1- and H5N1-infected P815 cells. (**A**) Heatmap of the Pearson correlation of each sample. The correlation coefficients can reflect the similar situation of the overall gene expression between each sample. The higher the correlation coefficient is, the more similar the gene expression level is. (**B**) Volcano plots of DEGs in H1N1_vs_Mock and H5N1_vs_Mock. (**C**) Bar chart showing the up-regulated DEGs and down-regulated DEGs in P815 cells infected with H1N1 and H5N1 virus. (**D**) Venn diagram showing the overlapping and specific DEGs of H1N1- and H5N1-infected P815 cells. (**E**) Heatmap of predominant up- and down-regulated DEGs in P815 cells infected with H1N1 and H5N1 virus. (**F**) The qRT-PCR analysis of the expression of HA gene in H1N1- and H5N1-infected P815 cells relative to the uninfected controls. Data are expressed as the mean ± SD. ***, *p* < 0.001. (**G**) The qRT-PCR analysis of randomly selected cytokine genes in H1N1- and H5N1-infected P815 cells relative to the uninfected controls. Data are expressed as the mean ± SD. ***, *p* < 0.001; #, *p* < 0.0001.

Samples collected from H1N1-infected, H5N1-infected and control mouse MC line P815 were subject to RNA-seq. Pearson correlation analysis among the samples revealed a high degree of correlation between biological replicates of the same condition (Figure 1A). Both H1N1 and H5N1 virus infection induced dynamic transcriptional changes in P815 cells, with features shared by both viruses or specific to either one (Figure 1B). 

H1N1 and H5N1 infection significantly activated the gene expression of cytokines and chemokines, such as Cxcl10, Il13, Ccl3, Ccl4 and Isg15. Interestingly, H1N1 infection down-regulated certain genes, such as Itgb3, Irf9, Hvcn1 and Nbeal2, which was not observed during H5N1 infection. To analyze the host immune responses that were common and specific to H1N1 and H5N1 virus infection, we compared the DEGs (including both up- and down-regulated genes) under each infection condition. Among all the DEGs identified in H1N1-infected P815 cells, 1792 were up-regulated, while 450 were down-regulated (Figure 1C). 

On the contrary, H5N1 infection changed the host transcriptome to a much lesser degree, with only 868 up-regulated genes and 217 down-regulated genes. There were 824 up-regulated genes and 136 down-regulated genes shared between H1N1- and H5N1-infected P815 cells (Figure 1D). In addition, 967 genes were up-regulated and 314 genes were down-regulated specifically during H1N1 infection. Interestingly, H5N1 virus induced a much smaller number of virus-specific DEGs with 44 up-regulated and 80 down-regulated genes. 

The heatmap of DEGs showed that H1N1 and H5N1 infection also triggered the expression of marker genes related to different biological events (Figure 1E). For example, H1N1 infection up-regulated interferon-inducible transmembrane (IFITM) genes and NADH dehydrogenase iron-sulfur protein related genes, while H5N1 infection induced higher levels of interleukins and chemokines. To validate our transcriptomic analysis, we also confirmed the expression level of the HA gene and several cytokine genes using qRT-PCR (Figure 1F,G). 

The transcripts of both HA gene and examined marker genes exhibited significant increases at different time points post-infection. Taken together, these results suggest that H1N1 infection led to a greater global change to the transcriptome of P815 cells than H5N1 infection. The shared DEGs between the two IAV strains largely overlapped with the DEGs identified from H5N1 infection, and their infection led to the expression of marker genes characterized to different pathways.

### 3.2. Gene Ontology Analysis of Mast Cells Infected with H1N1 and H5N1 Viruses

**Figure 2:**
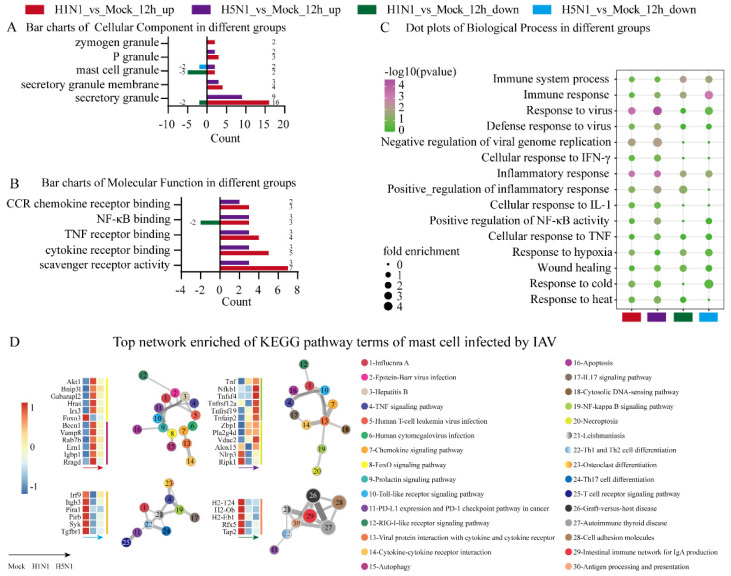
GO annotation and KEGG pathway enrichment analyses of DEGs in P815 cells infected with H1N1 virus and H5N1 virus. The online website g: Profiler was used for the functional enrichment analysis of DEGs. (**A**) Bar charts of cellular component terms of DEGs associated with mast cell granules in P815 cells infected with H1N1 virus and H5N1 virus. (**B**) Bar charts of molecular function terms related to chemokine and cytokine in P815 cells infected with H1N1 virus and H5N1 virus. (**C**) Dot plots of biological process terms of DEGs related to immune responses in P815 cells infected with H1N1 virus and H5N1 virus. (**D**) Top network enrichment of KEGG pathway terms of P815 cells infected with H1N1 and H5N1 virus. The network was visualized using Cytoscape, where each node represents an enriched term and is labeled by its cluster ID and *p*-value with a color.

To dive into the possible biological processes related to different viral infections, the above DEGs were analyzed using online tools. The DEGs in each group were first annotated and then subject to enrichment analysis. The enriched GO terms are shown in Figure 2. Enrichment analysis of up-regulated DEGs highlighted the CC GO terms, including the secretory granule, secretory granule membrane, mast cell granule, P granule and zymogen granule, during H1N1 and H5N1 infection (Figure 2A). 

MF GO term analysis showed that the up-regulated DEGs under H1N1 infection were more enriched in scavenger receptor activity, cytokine receptor binding, TNF receptor binding, NF-κB binding and CCR chemokine receptor binding compared with H5N1_vs_Mock (Figure 2B). BP GO term analysis showed that H5N1 infection was enriched for GO terms, including the response to virus, immune response, inflammatory response, positive regulation of NF-κB activity, response to cold and negative regulation of viral genome replication, compared with H1N1 infection, while H1N1 infection significantly induced the response to heat (Figure 2C). 

We also examined network enrichment in the DEGs to identify the statistically enriched KEGG pathways of MCs infected by the two IAV strains. The up-regulated genes in the H1N1-infected group were significantly enriched in the FoxO signaling pathway and autophagy, whereas the up-regulated genes in the H5N1-infected group were enriched in the NF-κB signaling pathway and necroptosis. On the contrary, the down-regulated genes in H5N1-infected group were enriched in osteoclast differentiation while the down-regulated genes in the H1N1-infected group were enriched in antigen processing and presentation. These results indicated the two IAV strains induced different signal transduction pathways and cell death pathways.

### 3.3. Comparison of H5N1- and H1N1-Infected Mast Cells

**Figure 3:**
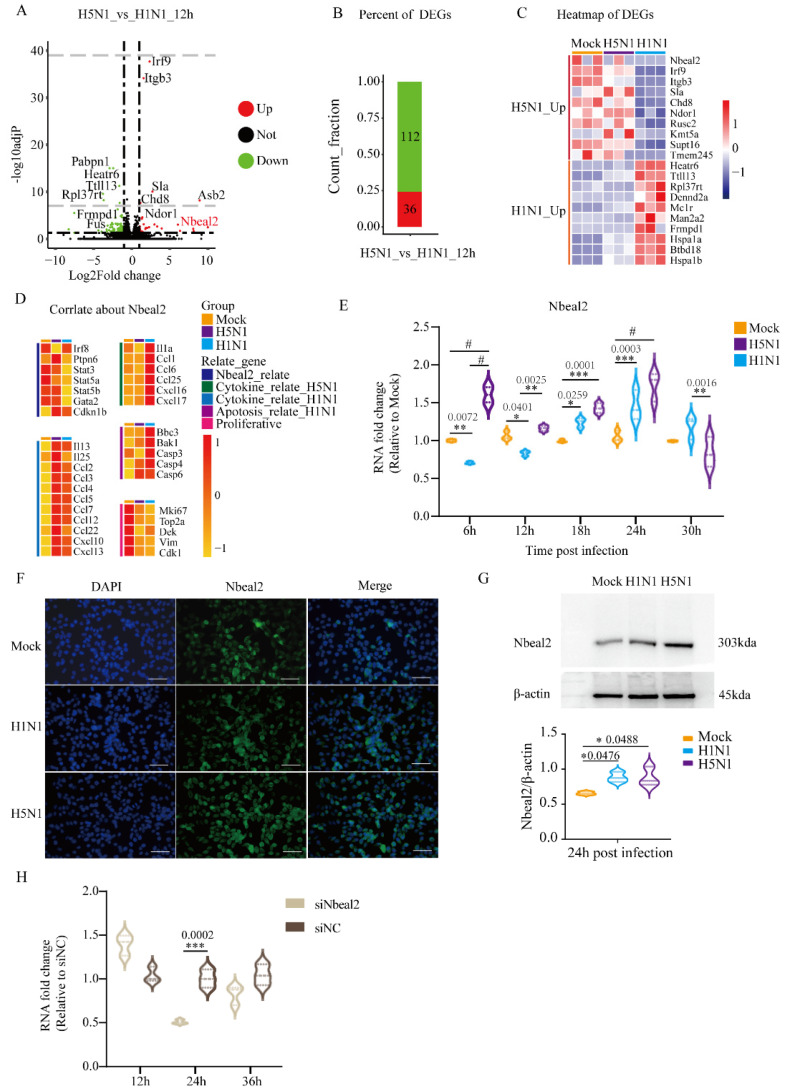
Analysis of DEGs in H5N1_vs_H1N1_12h and verification of Nbeal2 by qRT-PCR and immunofluorescent staining. The volcano plots and heatmaps of DEGs were achieved with the heatmap packages from R. (**A**) Volcano plots of DEGs in H5N1_vs_H1N1_12h. (**B**) Bar charts of DEGs in H5N1_vs_H1N1_12h. (**C**) Heatmap of the predominant up-regulated DEGs in H5N1 and H1N1-infected P815 cells. (**D**) Analysis of the correlation of the Nbeal2 gene with H5N1 and H1N1 infection. (**E**) The expression of the Nbeal2 gene in H1N1- and H5N1-infected P815 cells relative to the uninfected controls by qRT-PCR analysis. Data are expressed as the mean ± SD. *, *p* < 0.05; **, *p* < 0.01; ***, *p* < 0.001; #, *p* < 0.0001. (**F**) Immunofluorescence staining for the Nbeal2 gene at 24 h post-infection in P815 cells. (**G**) Western blot detected the expression of Nbeal2 at 24 h post-infection. Data were obtained from three independent replicates. (**H**) The Nbeal2 gene expression in P815 cells at the indicated time after siNbeal2 transfection. The transfection efficiency was measured by qRT-PCR. Data were obtained from three independent replicates. Data are expressed as the mean ± SD. ***, *p* < 0.001.

To further investigate the difference between H5N1 and H1N1 infection, we directly compared the transcriptomes of H5N1- and H1N1-infected cells and identified 36 up-regulated and 112 down-regulated DEGs (Figure 3A,B). The heatmap of DEGs shows specific 10 up-regulated DEGs of H5N1 infection and H1N1 infection (Figure 3C). As granules represented one of the previously identified CC GO terms and Nbeal2 has been implicated MC degranulation [23], we focused on the Nbeal2 gene for further analysis. The heatmap in Figure 3D shows the genes related to Nbeal2 and genes related to cytokines.

Consistent with the result of RNA-seq analysis, the Nbeal2 expression showed significant difference between H5N1- and H1N1-infected P815 cells at 12 h post-infection. We next followed Nbeal2 transcription in H5N1- and H1N1-infected cells every 6 h upon infection in details. Between 6 and 12 h post-infection, the Nbeal2 mRNA levels decreased in P815 cells infected with H1N1 virus, while they significantly increased in those infected with H5N1. From 18 to 24 h post-infection, the mRNA levels of Nbeal2 increased in P815 cells infected with either H1N1 or H5N1 virus. Meanwhile, H5N1-infected P815 cells showed significantly higher Nbeal2 mRNA levels compared with H1N1-infected P815 cells. 

The Nbeal2 mRNA expression in P815 cells infected with H1N1 or H5N1 virus peaked at 24 h post-infection before decreasing (Figure 3E). Consistent with our qRT-PCR results, immunohistochemistry and western blotting using an antibody against Nbeal2 at 24 h post-infection further verified the increased expression of Nbeal2 in H5N1-infected P815 cells. The results indicated that Nbeal2 was expressed at a higher level in H5N1-infected P815 cells compared with in H1N1-infected P815 cells (Figure 3F,G). Taken together, Nbeal2 may play an important role during H1N1 and H5N1 infection, especially in H5N1 virus infection.

### 3.4. Effect of Nbeal2 on the Inflammatory Response Following H1N1 and H5N1 Virus Infection

To further identify the functional relevance of Nbeal2 in the cell response, P815 cells were transfected with three mixed small interfering RNA sequences to knockdown Nbeal2 expression before they were infected with H1N1 and H5N1 influenza viruses (Figure 3H). To further confirm the role of Nbeal2 in the cellular antiviral response, we next explored the expression of cytokines that regulate the inflammatory responses induced by H1N1 and H5N1 viruses. 

**Figure 4:**
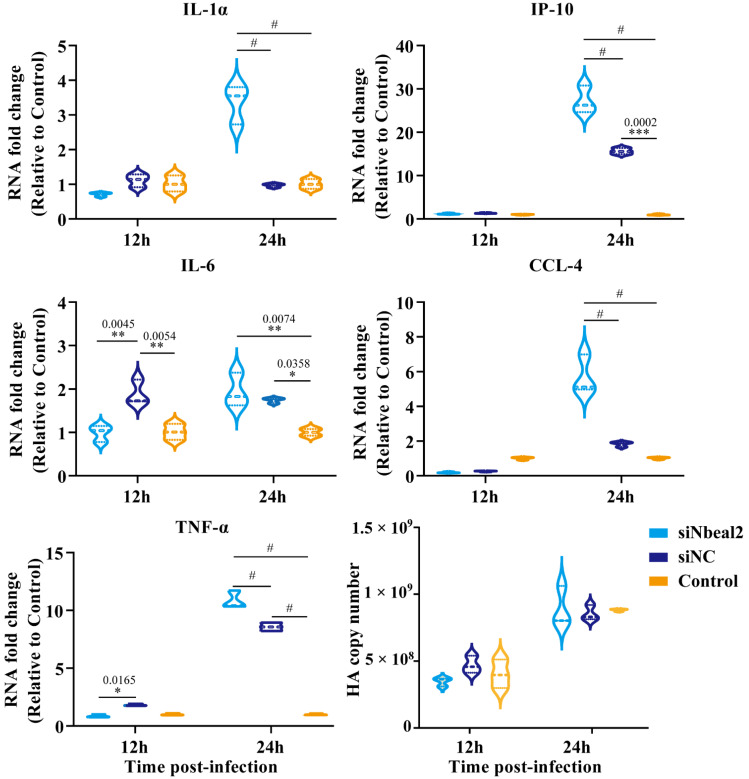
Cytokine expression analysis of H1N1-infected P815 cells with siNbeal2 transfection by qRT-PCR. P815 cells were transfected with siNbeal2 and were then infected with H1N1 virus. The expression of IL-1α, IP-10, IL-6, CCL-4 and TNF-α and the HA copy number were measured at 12 h and 24 h post infection using qRT-PCR. Control represents the group with H1N1 virus challenge without siNbeal2 transfection. The results shown were obtained from three independent replicates. Data are expressed as the mean ± SD. *, *p* < 0.05; **, *p* < 0.01; ***, *p* < 0.001; #, *p* < 0.0001.

**Figure 5:**
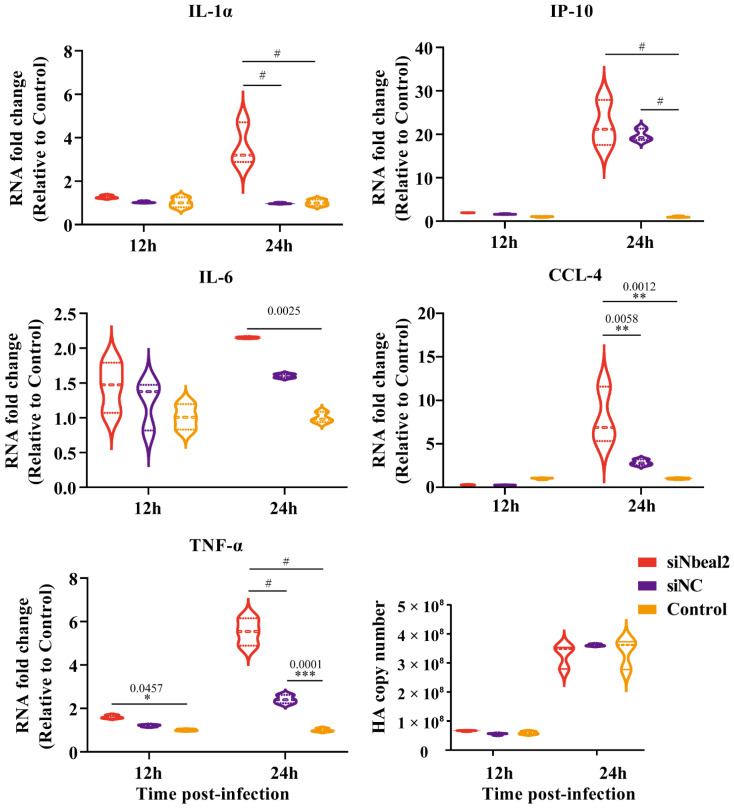
Cytokine expression analysis of H5N1-infected P815 cells with siNbeal2 transfection by qRT-PCR. P815 cells were transfected with siNbeal2 and were then infected with H5N1 virus. The expression of IL-1α, IP-10, IL-6, CCL-4 and TNF-α and the HA copy number were measured at 12 h and 24 h post infections by qRT-PCR. Control represents the group with H5N1 virus challenge without siNbeal2 transfection. The results shown were obtained from three independent replicates. Data are expressed as the mean ± SD. *, *p* < 0.05; **, *p* < 0.01; ***, *p* < 0.001; #, *p* < 0.0001.

Remarkably, the knockdown of Nbeal2 significantly promoted the production of IL-1α, IP-10, IL-6, CCL-4 and TNF-α at 24 h post-infection in P815 cells infected with H1N1 virus and H5N1 virus. The knockdown of Nbeal2 did not affect the viral replication of P815 cells infected with H1N1 virus and H5N1 virus. (Figure 4 and Figure 5). These results suggested that Nbeal2 could directly inhibit inflammatory cytokine release without affecting viral replication. To investigate whether the activated cytokines released by IAV infected-P815 cells could trigger pulmonary epithelial cell apoptosis, we used the supernatant of the infected P815 cells to culture A549 for 24 h and analyzed the apoptosis rate using flow cytometry. 

**Figure 6:**
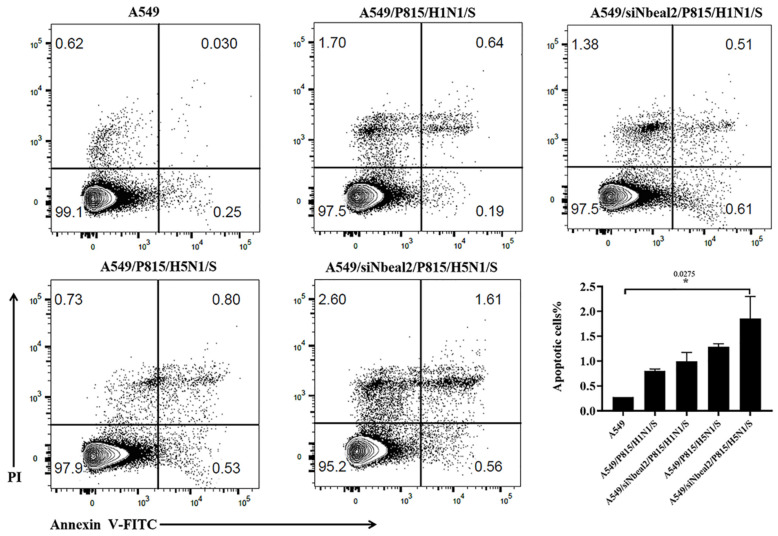
Apoptosis rates of A549 cells cultured by supernatants of IAV-infected P815 cells. Apoptosis rates were analyzed by FACS using the FITC Annexin V Apoptosis Detection Kit. *, *p* < 0.05, n = 2. P815/H1(5)N1/S: supernatant from H1(5)N1 infected-P815 cells; siNbeal2 /P815/H1(5)N1/S: supernatant from H1(5)N1 infected-P815 cells with Nbeal2 knock down; and S: supernatant.

The supernatant collected from the infected, Nbeal2-knockdown P815 cells (Figure 6, A549/siNbeal2/P815/H1N1/S and A549/siNbeal2/P815/H5N1/S) led to increased apoptosis in A549 cells compared with the control infected cells (A549/P815/H1N1/S and A549/P815/H5N1/S). Importantly, the H5N1-infected, Nbeal2-knockdown cells’ supernatant had the strongest effect in inducing cell apoptosis (Figure 6, A549/siNbeal2/P815/H5N1/S). These results suggest that Nbeal2 can regulate the production of cytokines induced by H5N1 and H1N1 viruses in MCs and that the knockdown of Nbeal2 resulted in increased cytokine release, which caused increased apoptosis.

## 4. Discussion

The latest progress in biomedical research benefits from the recent technological advancement in technologies, which allows the systemic investigation of genomic structures and cellular, physiological and biochemical responses to diseases and pathogenic perturbations. Here, next generation sequencing (RNA-seq) is a state-of-the-art approach that offers nearly unlimited possibilities in modern research [24,25]. Our previous study employed microarray techniques to characterize the dynamic genomic profiles of mouse MCs modulated by influenza A viruses H1N1, H5N1 and H7N2 [26]. 

The present study aimed to further create comprehensive and comparative transcriptional profiles of MCs responding to IAV infection. Using RNA-seq, we first analyzed differences between H5N1 virus and H1N1 virus. We successfully identified 2242 and 1085 DEGs in mouse MCs (P815 cells) infected with either virus, while our previous study identified 216 and 101 DEGs in MCs (P815 cells) infected with H1N1 virus and H5N1 virus, respectively [26]. Moreover, by performing GO term and KEGG pathway enrichment analyses, we sought to explore the underlying molecular mechanisms by which host defense systems respond to IAV infection in MCs. 

Here, the top network-enriched KEGG pathways showed distinct differences between H1N1 virus and H5N1 virus infection in mouse mast cells. H1N1 virus infection more intensely activated pathways, such as the foxO signaling pathway and autophagy pathway, compared with H5N1 virus infection, suggesting that the mechanisms in these signaling pathways might be associated with varying subtypes of IAVs. Therein, consistent with our previous analysis that the foxO pathway plays a significant role in IAV infection through mediating the anti-apoptosis and anti-inflammatory responses [27], this may help to explain why H5N1 virus infection typically leads to more intense damage compared with H1N1 virus infection. 

H5N1 virus infection dramatically enriched NF-κB, which is known as a major regulator of inflammatory response and necroptosis pathway. Our results were consistent with the previous research that low pathogenic influenza strains revealed a much weaker and less NF-κB-dependent host cell response compared with H5N1 virus [28]. However, more than that, necroptosis pathways were newly discovered. One strength of the current study is that we specifically assessed transcriptomic dynamics and cellular functions related to the specific degranulation of MCs as well as the inflammatory and immune responses of MCs infected by two subtypes of IAVs. 

The results showed that H5N1 infection appeared to activate MCs more intensely than H1N1 infection. We next analyzed the transcriptomes pf H5N1- and H1N1-infected MCs in parallel and found that many specific genes were differentially expressed among which Nbeal2 stood out. We prioritized Nbeal2, as it has been previously associated with MC degranulation [29].

Nbeal2 belongs to a gene family involved in granule development [29]. Mutations in the human Nbeal2 gene can cause gray platelet syndrome (GPS), a bleeding 85 diathesis characterized by a lack of α granules in platelets [30]. A previous study demonstrated that human megakaryocytes and platelets express a unique combination of Nbeal2 isoforms and that Nbeal2 localizes to the endoplasmic reticulum in platelets [23]. The functions of the Nbeal2 gene have been poorly explored outside platelet biology. Recent research has shown anomalies in the function of neutrophils and natural killer (NK) cells and impaired host defense after infection of Staphylococcus aureus in Nbeal2-deficient mice [31,32]. 

Furthermore, Nbeal2 has also been reported to mediate granule formation and the effector function of MCs [33]. These data together strongly suggest a plausible role of Nbeal2 in the immune response. However, the role of Nbeal2 in MCs during IAV infection and its potential mechanisms remain to be determined. In the present study, we first established the role of Nbeal2 in the innate response during IAV infection by examining the expression of Nbeal2 in P815 cells infected with H1N1 and H5N1 viruses. The mRNA levels of Nbeal2 were consistent with our transcriptome analysis.

Next, we explored the role of Nbeal2 in the immune response. MCs have been reported to play a key role during IAV infection [34,35]. MCs are involved in enhancing IAV-mediated disease and release disease-causing mediators, including TNF-α, IL-6 and IL-1 [36]. Our previous study suggested that activated MCs can induce apoptosis of the pulmonary epithelial cells by releasing proinflammatory cytokines [26]. The results from the current study showed that P815 cells in which Nbeal2 was knocked down exhibited significantly increased expression levels of IL-1α, IP-10, IL-6, CCL-4 and TNF-α during both H1N1 and H5N1 virus infection, indicating that Nbeal2 plays a critical role in inducing the activation of MCs. 

Some studies have suggested that Nbeal2 controls the release of cytokines by regulating the granule formation [37]. We therefore confirmed that the enhanced cytokines released by IAV-infected P815 cells could induce A549 cell apoptosis, and the effect was further reinforced with Nbeal2 knockdown. Here, we showed that Nbeal2 played an essential role in the immune response caused by H5N1 and H1N1 virus infection. Nbeal2 knockdown triggered more intense cytokine production of MCs in H5N1 infection compared with H1N1 infection, and the mediators further enhanced A549 cell apoptosis. 

Expectedly, Nbeal2 knockdown did not directly affect the viral replication of IAV-infected P815 cells, mainly due to mast cells acting primarily as inflammatory cells triggering a pro-inflammatory response during IAV infection rather than as target cells for viral replication [31,32,33]. Altogether, our results indicated that Nbeal2 played significant roles in the immune regulation of MCs in response to IAV infection, and the effect of Nbeal2 was more prominent during H5N1 infection, which might be related to the cytokine storm triggered by H5N1 infection.

In summary, for the first time, we established the roles of Nbeal2 in two IAV strain infections and the crosstalk between Nbeal2 regulation by IAV infection as well as its effect on innate immunity in MCs, which suggested that Nbeal2 may be a promising target for future therapeutic interventions.

## Figures and Tables

**Table 1:** Gene sequences.

Gene	Sequences
Nbeal2-Mus-1177	F: 5′-GCUACAAGCCACCUUCCUUTT-3′
	R: 5′-AAGGAAGGUGGCUUGUAGCTT-3′
Nbeal2-Mus-3274	F: 5′-GCACCUGCUCUUCAACUUUTT-3′
	R: 5′-AAAGUUGAAGAGCAGGUGCTT-3′
Nbeal2-Mus-8095	F: 5′-GCACCUGUAUUCAGUGAAUTT-3′
	R: 5′-AUUCACUGAAUACAGGUGCTT-3′
negative control	F: 5′-UUCUCCGAACGUGUCACGUTT -3′
	R: 5′-ACGUGACACGUUCGGAGAATT -3′

## Data Availability

Research data pertaining to this article can be found in the NCBI using BioProject ID: PRJNA745454.
